# Redox and solvent-stable alkaline serine protease from *Bacillus patagoniensis* DB-5: heterologous expression, properties, and biotechnological applications

**DOI:** 10.3389/fmicb.2025.1558419

**Published:** 2025-03-19

**Authors:** Zhimin Li, Yanmin Xing, Pulin Liu, Weifang Liao, Lihong Miao

**Affiliations:** College of Life Science and Technology, Wuhan Polytechnic University, Wuhan, China

**Keywords:** alkaline protease, gene expression, *Bacillus patagoniensis* DB-5, enzymatic properties, detergent bio-additive, feather degradation, whey protein hydrolysis

## Abstract

The *aprBP* gene from *Bacillus patagoniensis* DB-5, encoding a 378-amino-acid alkaline protease, was cloned and expressed in *Escherichia coli*. The amino acid sequence of APrBP showed 62.8–84.4% identity with the S8 peptidase subtilisin family alkaline proteases reported in the literature. Recombinant APrBP was purified using Ni-NTA affinity chromatography with 45.61% recovery and a homogeneous band was detected at approximately 38 kDa on the SDS-PAGE gel. The optimum temperature of APrBP was 60°C. The presence of 2 mM Ca^2+^ significantly enhanced the optimal temperature and thermostability. The enzyme demonstrated optimum activity at pH 12 and maintained high stability at pH 8.0–11.0. Protease activity was stimulated by Mn^2+^, Ca^2+^, Mg^2+^, Ni^2+^, TritonX-100, Tween-20 and Tween-80, while completely inactivated by PMSF, EDTA and Cu^2+^. The APrBP exhibited good tolerance to oxidizing and reducing agents. Notably, the protease exhibited remarkable stability in 50% (v/v) concentrations of several organic solvents, such as methanol, acetone, glycerol, dimethyl sulfoxide, n-hexane, and ethyl acetate. The APrBP efficiently hydrolyzed natural proteins, demonstrating the highest catalytic efficiency for casein, excellent hydrolysis activity for bovine serum albumin, hemoglobin, and keratin, and favorable hydrolysis ability for whey proteins. Moreover, molecular docking results revealed stable interactions between APrBP and casein, hemoglobin, whey proteins and keratin. This study indicated that APrBP has some useful properties and explored its potential as a bio-additive detergent as well as in utilizing feather waste and whey protein.

## 1 Introduction

As one of the major groups of industrial enzymes, proteases are hydrolases responsible for the breakdown of polypeptides or proteins (Contesini et al., 2018). Alkaline proteases exhibit maximum activity at pH 8.0–12.0, typically belonging to serine protease and metalloprotease family (Song et al., 2023). Alkaline proteases can be produced by microorganisms, plants, and animals. However, those derived from microorganisms are preferred because of their high yield, good stability, strong specificity, and ease of genetic manipulation (Sarwa et al., 2024).

Numerous proteases have been isolated and characterized from *Bacillus* strains which are well known for producing various extracellular enzymes. Different alkaline proteases have been derived from *Bacillus* species, including *Bacillus subtilis* (Rai and Mukherjee, 2009), *Bacillus licheniformis* (Hadjidj et al., 2018), *Bacillus amyloliquefaciens* (Guleria et al., 2016), haloalkaliphilic *Bacillus* sp. (Dodia et al., 2008), *Bacillus cereus* (Zambare et al., 2007), *Bacillus lentus* (Jørgensen et al., 2000) and *Bacillus circulans* (Chen et al., 2022). Whereas, there remains a need for more alkaline proteases that function well under stress conditions.

To address the growing demand for proteases capable of performing effectively under extreme processing conditions, researchers have embarked on numerous explorations. Isolating extremozymes from extreme environments is a promising avenue for obtaining premium enzymes. For example, the thermostable protease from the thermophilic bacterium, *Bacillus stearothermophilus* TLS33 retained 31% of its enzymatic activity after incubation at 100°C for 30 min (Sookkheo et al., 2000). Alkaline proteases from haloalkaliphilic microorganisms exhibit excellent characteristics under extreme conditions of high alkalinity, elevated salt levels and substantial amounts of organic solvents, highlighting their significance for the industry (Doukyu and Ogino, 2010). For instance, solvent-stable proteases could be used in peptide synthesis. The protease from *Bacillus cereus* WQ9-2 synthesized the precursor (Cbz-Ala-Phe-NH2) of a bitter dipeptide in the presence of 50% dimethyl sulfoxide (DMSO) (Xu et al., 2010). The PST-01 protease from *Pseudomonas aeruginosa* demonstrated high efficiency in synthesizing the aspartame precursor (cbz-asp-phee-ome) in aqueous organic solvents (Tsuchiyama et al., 2007). Therefore, it is of great importance to explore more extremophilic alkaline proteases to meet various industrial applications.

Alkaline proteases have widespread applications in industries, including leather, detergents, cosmetics, food, pharmaceuticals, and waste treatment (Mokashe et al., 2018). Currently, proteases are commonly incorporated into detergents due to their ability to remove protein-based stains and improve cleaning efficiency. However, proteases should be robust to be compatible with the oxidizing reagents, surfactants, high salt concentrations, and alkaline environments while also maintaining high activity at 20–60°C (Hadjidj et al., 2018). The search for highly active and detergent-stable proteases from haloalkaliphilic microorganisms is of great interest in the industrial sector.

Recently, the utilization of feather waste has garnered increasing attention in the field of waste treatment. With the expansion of the global poultry production market, the amount of poultry waste has reached 68 billion tons, with feathers accounting for 5–10% of the total weight of poultry animals (El Salamony et al., 2024; Liang et al., 2021). The inadequate management of poultry feather waste has caused serious environmental challenges (Das et al., 2024). Nevertheless, the high protein content of poultry feathers facilitates for feather utilization, enabling their conversion into soluble proteins and functional amino acids, thereby promoting circular economic utilization (Grazziotin et al., 2006). Enzymatic digestion of feathers is an eco-friendly method that yields higher products than acid or alkaline treatments. Nevertheless, most feather hydrolyzing proteases have low enzymatic activity and limited processing capability (Verma et al., 2017). Thus, it is crucial to explore additional candidate enzymes to provide insights for practical applications.

Whey protein is a portion of milk protein that remains soluble after coagulation by rennet or precipitation by acid, and primarily consists of β-lactoglobulin (β-LG), α-lactalbumin (α-LA) and bovine serum albumin (BSA) (Lu et al., 2022; Sharma et al., 2019). As a by-product of dairy production, whey protein serves as both an environmental challenge and a low-cost resource (Zandona et al., 2021). Enzymatic hydrolysis, requires milder reaction conditions and is an eco-friendly approach, compared to chemical and physical methods. Commercial proteases are currently dominantly used for whey protein hydrolysis, however, the use of non-commercial proteases, especially those derived from haloalkaliphiles, has rarely been reported (Lermen et al., 2023).

In this context, we obtained a haloalkaliphilic *Bacillus patagoniensis* from saline-alkali soil, exhibiting alkaline protease activity in its culture supernatant. Although the alkaline proteases secreted by *Bacillus patagoniensis* have been characterized (Olivera et al., 2006), the corresponding genes have yet to be cloned and expressed, leaving their commercial potential largely unexplored. The sequence information of the protease is crucial for understanding how its structure determines its function. What’s more, the commercialization of any enzyme requires large-scale production, that can be achieved through heterologous gene expression. The aim of this study was to express a new alkaline protease derived from *Bacillus patagoniensis* for exploring its biotechnological potential. In this study, the recombinant APrBP was overexpressed in *Escherichia coli*, followed by purification and biochemical characterization. Furthermore, its potential applications as detergent additives, in feather degradation and whey hydrolysis were thoroughly evaluated.

## 2 Materials and methods

### 2.1 Strains, plasmids and culture conditions

*Bacillus patagoniensis*, a haloalkaliphilic strain derived from saline-alkali soil of Jiamusi, Heilongjiang Province, China, was used as the source of the *aprBP* gene. The pET28a (+) vector was chosen for protein expression. *E. coli* DH5α and *E. coli* BL21 (DE3) were employed as storage and expression strains, respectively, and were cultured in Luria-Bertani broth (LB) media. If necessary, kanamycin (50 μg/mL) was supplemented into the medium.

### 2.2 Cloning and sequence analysis of APrBP gene

Genomic DNA was extracted from 2 mL bacterial culture using a QIAamp DNA Kit (Qiagen). Based on the sequence of the *Bacillus patagoniensis* genome (GenBank Accession No. GCA_002019705.1) from the NCBI database, the protease gene *aprBP* was identified using the ORF finder tool on NCBI. A set of primers APrBP *BamHI* F′ (5′-GGGTCGCGGATCCGAATTCGCAGAGGAAGCGAAAGAG-3′) and APrBP *NotI* R′ (5′-CGAGTGCGGCCGCAAGCTTACGTGT TGCTGCTTCTGCG-3′) with BamHI and NotI restriction site were designed to amplify the APrBP gene without signal peptide. The target fragment was successfully amplified using the genomic DNA of *Bacillus patagoniensis*. The PCR product was purified and ligated into pET28a (+) using Gibson assembly (NEB). After being verified by sequencing, the sequence alignment was performed by NCBI blastn.

The signal peptide was analyzed to predict the cleavage site using the SignalP 6.0 server (Teufel et al., 2022). The theoretical isoelectric point and molecular weight were determined using the Expasy website. The NCBI Conserved Domain Search is used to obtain the catalytic sites and the active sites. Alignment of multiple sequences were conducted by ClustalW 2.0, and illustrated by Endscript 2.0. Expasy’s Protparam server was used for prediction of primary structures of APrBP and other alkaline proteases. The phylogenetic tree was constructed using the neighbor-joining method in MEGA 11 software (Tamura et al., 2021).

### 2.3 Structural modeling

The Swiss-model server was utilized to identify the template and generate the three-dimensional (3D) structure of the APrBP mature protein. The 3D protein model was analyzed and visualized by Pymol 2.6 program. Furthermore, the quality of the generated model was evaluated using several protein assessment tools including the ProSA, PROCHECK, VERIFY3D and ERRAT program.

### 2.4 Molecular docking

The previous modeled 3D structure of APrBP was docked to the substrates. Molecular docking between the protease APrBP and the small molecule ligands was simulated using Autodock 4.2. Considering the alkaline protease’s recognition of hydrophobic amino acids and positively charged amino acids, the following peptides were selected for simulation experiments: casein (UniProt ID: P02666, sequence QAFLLYQEPVL), keratin (PubChem CID: 71464397, sequence LNDRKASYL), α-lactalbumin (UniProt ID: P00711, sequence FHTSGYDTQAI), β-lactoglobulin (UniProt ID: P02754, sequence DTDYKKYLLF), BSA (PDB ID: 4F5S, BSA, sequence HPEYAVSVL), and hemoglobin (PubChem CID: 13285535).

The 3D structures of the selected peptides were constructed using ChemDraw 3D and exported in the PDB format. The docking results were visualized using PyMOL and MOE2019 (for academic use only), displaying the binding modes of the substrate peptides around the active sites of the alkaline protease.

### 2.5 Protease activity assay and protein concentration determination

The enzyme activity was detected by using casein as the substrate, according to the Folin–Ciocalteu method of the People’s Republic of China Standard GB/T 23527-2009. The blank control was treated the same as the experimental group, except that TCA was added before the enzyme. One unit of proteolytic activity was defined as the quantity of protease required to generate 1 μg of tyrosine per minute. The protein concentration was determined using the Bradford Protein Assay Kit (Solarbio, China) (Bradford, 1976).

### 2.6 Expression, purification and identification of APrBP

The recombinant vector pET-28a (+)-*aprBP* was transformed into *E. coli* BL21 (DE3). A positive APrBP-pET28a colony was cultured in LB medium with kanamycin (50 μg/mL), then inoculated into 200 mL LB medium (2% v/v) with the same antibiotic concentration, and incubated at 37°C until the optical density reached 0.5–0.6 at 600 nm (Lozano Terol et al., 2021). The bacteria were incubated for 24 h at 16°C, followed by the addition of0.1 mM isopropyl β-D-1-thiogalactopyranoside (IPTG). Cells were collected by centrifugation at 8,000 g for 30 min and washed with lysis buffer (50 mM phosphate buffer and 300 mM NaCl). The suspension was sonicated for 40 min on ice. The supernatant was obtained after centrifugation (12,000 g, 10 min) for purification. The Ni-NTA chromatography column was connected to a peristaltic pump, and the liquid flow rate was set at 1 mL/min. The column was washed with ultrapure water for 20 min to remove any residual ethanol. The column was equilibrated with lysis buffer for 10 min. The supernatant was added to the column multiple times to facilitate the binding of the recombinant APrBP to the Ni-NTA resin. The column was washed with a washing buffer (lysis buffer and 20 mM imidazole) for 10 min to remove non-specific proteins. Recombinant APrBP was eluted using elution buffers (lysis buffer and imidazole) at a linear gradient of 40–200 mM imidazole (Healthcare, 2007). The eluted fractions were checked for enzyme activity, and the purified fractions were further analyzed by 12% sodium dodecyl sulfate-polyacrylamide gel electrophoresis (SDS-PAGE) (Laemmli, 1970). The purified protein APrBP was analyzed using UltiMate 3000 RSLCnano system coupled online with Q Exactive HF mass spectrometer through a Nanospray Flex ion source (Thermo). Sequence alignment with the *Bacillus patagoniensis* proteomics facilitated the identification of the complete protein sequence. Protein concentrations were assessed as previously described in this study.

### 2.7 Determination of biochemical properties

#### 2.7.1 Effects of temperature and pH on APrBP activity and stability

The optimal temperature was evaluated over 20–70°C range, under the condition mentioned in section 2.5. Thermal stability was determined by pre-incubating at 50, 55, and 60°C for a range of times (0.5 h to 6 h) and measuring the enzyme activity. Particularly, 2 mM CaCl_2_ was supplied to the enzyme solution to determine the effects of Ca^2+^ on optimum temperature and thermostability (Jeong et al., 2018) The optimal pH was analyzed using phosphate buffer (pH 6–7), Tris-HCl buffer (pH 8.0–9.0), and borate-NaOH buffer (pH 10.0–13.0) (Yang et al., 2020). pH stability was determined by incubating at each pH (8–13) at 25°C for 60 h. Aliquots were then taken out at regular intervals to determine the remaining enzyme activities.

#### 2.7.2 Effects of metal ions on APrBP activity

After maintaining the enzyme in the buffers containing metal ions including 5 and 10 mM of Ca^2+^, Ba^2+^, Mg^2+^, Mn^2+^, Ni^2+^, Fe^3+^, Fe^2+^, Cu^2+^, Co^2+^, and K^+^ at 25°C for 1 h, the remaining enzymatic activity was assessed as previously described, with the activity in the absence of metal ions defined as 100% (Yang et al., 2020).

#### 2.7.3 Effects of chemical reagents on APrBP activity

The enzyme was preincubated with surfactants (TritonX-100, Tween-80, Tween-20 and SDS), inhibitors (EDTA and PMSF), oxidant and reducing agents (H_2_O_2_, β-mercaptoethanol and DTT) and NaCl (0.5, 1, and 2.5 M) for 1 h at 25°C. The residual enzyme activity was measured under standard conditions and the enzyme activity without additives was taken as 100% (Bhatt and Singh, 2020).

#### 2.7.4 Effects of solvents on APrBP stability

The enzyme was mixed with 50% (v/v) of various organic solvents including methanol, acetone, glycerol, DMSO, n-hexane, ethyl acetate, and chloroform, and incubated at 25°C for 72 h with shaking at 150 rpm (Jain et al., 2012; Xu et al., 2010). The residual enzyme activity was assayed under standard conditions. A control was preincubated without organic solvents was taken as 100%.

### 2.8 Substrate specificity and kinetic studies

The substrate specificity was determined by using casein, BSA, hemoglobin, azo-casein, whey protein, keratin, soybean protein, cottonseed protein, skimmed milk, gelatin and collagen at 2% concentration as substrates (Jeong et al., 2018) and the enzyme activity was measured as previously described in this study.

Enzyme activity was measured at different concentrations of casein substrate (1–20 mg/L) under an optimal condition (Yang et al., 2020). *V*_max_ (maximal velocity) and *K*_m_ (Michaelis–Menten constant) were evaluated based on the Lineweaver–Burk plot, using the Graphpad Prism12.0 software. *K*_cat_ (catalytic efficiency) was calculated using the following formula:


$$K_{\mathrm{cat}}=V_{\text{max}}\cdot {\left[E_{t}\right]}^{-1}\text{,}$$

where [*E_t_*] is the enzyme concentration.

### 2.9 Applications of the recombinant APrBP

#### 2.9.1 Analysis of stability and washing performance in detergent

APrBP was added to Tide detergent solutions at different ratios [enzyme: detergent (v/v) = 1:2, 1:5 and 1:10] and incubated at 40°C for 30 min (Jeong et al., 2018). The residual enzyme activity was then measured. The stability of APrBP was determined by comparing it to the commercial protease Savinase^™^.

The washing ability of APrBP was evaluated using new cotton cloth pieces (3 × 3 cm), stained with 0.25 mL of goat blood. The stain was dried for 24 h, and washed with 25 mL of tap water containing 7 mg/mL heat-treated detergent and 50 U/mL of APrBP. The mixture was then shaken at 100 rpm for 10 min at 40°C. The controls were included tap water, non-heat treated detergent, and heat-treated detergent. The performance of the APrBP was evaluated visually (Hammami et al., 2017).

#### 2.9.2 Analysis of feather degradation

Chicken feathers were obtained from the local market, were washed several times by tap water and dried at 65°C for 48 h. Subsequently, 1% (w/v) feathers were placed in feather both (FB) containing 0.3% K_2_HPO_4_, 0.4% KH_2_PO_4_, and 0.5% NaCl and autoclaved at 121°C for 20 min. APrBP (1,000 U) was added to 50 mL of FB medium and incubated at 50°C for 48 h. The feathers hydrolysate was regularly taken out to determine feather degradation rate and soluble protein concentration in fermentation solutions.

The feather degradation rate was calculated using the weight loss method. The FB medium was filtered through filter paper to separate the residual feathers from the supernatant. The residual feathers were dried at 65°C and weighed. The degradation rate was calculated as the percentage of weight loss compared to the original feather weight (Liang et al., 2021).

To analyze the free amino acid composition and content in the FB medium after incubation, feather hydrolysate was precipitated with a 5-fold volume of 5% sulfosalicylic acid for 2 h at 4°C, followed by centrifugation (12,000 g, 20 min). The supernatant was filtered through a 0.2 μm membrane filter, and then analyzed using an L-8900 automatic amino acid analyzer (Liang et al., 2021). The soluble protein concentration was determined as described before in this study.

#### 2.9.3 Analysis of hydrolysis effects of whey protein

Whey protein was prepared at a 10% (w/v) concentration in NaOH-borax buffer (pH 10.5). The whey solution was treated with APrBP at 300 U (Bhatt and Singh, 2020), maintaining an enzyme-to-whey protein ratio of 8:100 (v/v) (Morais et al., 2013), and incubated at 50°C for 2 h with shaking at 100 rpm. The aliquots were taken out at regular intervals. Two negative controls were set as follows: the enzyme was replaced with borax-NaOH buffer and heat-treated APrBP.

Total protein was analyzed before and after the treatment, as previously described (Bradford, 1976). The molecular weight distribution of the degraded whey proteins was visualized using 15% SDS-PAGE.

#### 2.9.4 Measurement of whey protein hydrolysate antioxidant activity

The antioxidant activity was determined using Re’s et al. (1999) method. The same volume of distilled water as the hydrolysate was used as the blank. The ABTS radical scavenging rate (%) was estimated using the following equation:


$$\mathrm{ABTS radical scavenging rate}\;\left(\%\right)=\left(A_{\mathrm{blank}}-A_{\mathrm{sample}}\right)/A_{\mathrm{blank}}\times 100$$

The ABTS radical scavenging rate of the unhydrolyzed sample was taken as the control.

### 2.10 Statistical analysis

All experiments were performed in at least triplicate with the controls under the identical conditions. Statistical analysis of the original data was conducted with Graphpad Prism 10.2 software. The results were compared using Student’s *t*-test and were presented as the mean values ± standard deviation.

## 3 Results and discussion

### 3.1 Molecular analysis

**Figure 1:**
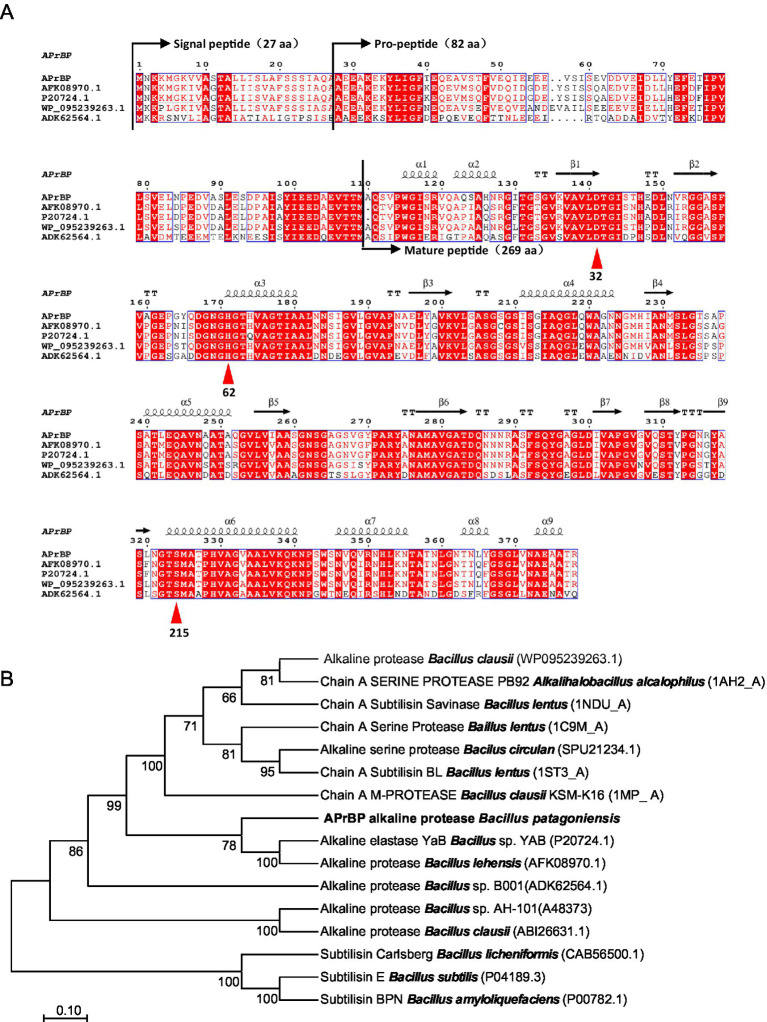
Alignment of amino acid sequences and phylogenetic tree of protease APrBP with those of other proteases. **(A)** Multiple sequence alignment of APrBP with other alkaline proteases: AFK08970.1, alkaline protease from *Bacillus lehensis*; P20724.1, alkaline protease from *Bacillus* sp. YAB; WP 095239263.1, alkaline protease from *Bacillus clausii*; ADK62564.1, alkaline protease from *Bacillus* sp. B001. The secondary structures are depicted at the top. Squiggles, arrows, and “T” represent the helices, β-strands, and β-turns, respectively. Similar sequences are indicated with a colored background, and the catalytic residues of D32, H62 and S215 are marked with up triangles. **(B)** Phylogenetic analysis of APrBP (black front) with other related enzymes based on the comparison of amino acid sequences. The neighbor-joining phylogenetic tree was constructed using MEGA 11 software. The values on the tree represent the percentages of bootstrap support derived from 1,000 replications. The scale bar corresponds to 0.1 change per amino acid.

The *aprBP* gene from a haloalkaliphilic bacterium *Bacillus patagoniensis*, containing an open reading frame of 1,134 bp, encoding 378 amino acids and one stop codon. The first 27 amino acids of APrBP represent the signal peptide, followed by the pro-peptide and mature peptide sequences from Ala28 to Met109 and Ala110 to Arg378, respectively (Figure 1A). The ExPASY software predicted that APrBP, excluding the signal peptide, has a molecular weight of 36017.65 Da and an isoelectric point of 4.53. The conserved domain spans from Gly134 to Thr356 while the catalytic triad consisting of Asp32, His62 and Ser215 (Figure 1A), as identified by NCBI CDD search analysis. The gene sequence encoding the APrBP pro-peptide and mature peptide has been submitted to the GenBank database (Accession No. PV031685).

BLASTP analysis indicated that the protein sequence of APrBP shares high similarity with proteins belonging to the S8 peptidase_subtilisin family. Specifically, it showed the highest similarity (99%) to the protease from *B. patagoniensis* (WP_078392427.1), which has only been annotated. Subsequently, the amino acid sequence exhibited 84.4% similarity to the protease from *Bacillus clausii* (WP_095239263.1), 83.1% similarity to the protease *Bacillus lehensis* (AFK08970.1) and the protease from *Bacillus* sp. YAB (P20724.1), and 62.8% similarity to the protease from *Bacillus* sp. B001 (ADK62564.1) (Figure 1A).

The neighbor-joining phylogenetic tree (Figure 1B) revealed that APrBP was placed in a cluster containing the alkaline proteases from *Bacillus lehensis* (AFK08970.1) and *Bacillus* sp. YAB (P20274.1), indicating a close evolutionary relationship with these two enzymes, followed by a close correlation with alkaline protease from *Bacillus clausii* (WP_078392427.1). This is in contrast to the similarity results from BLASTp, possibly due to the different data processing by the neighbor-joining method. APrBP is closely associated with Savinase from *Bacillus lentus*, but distantly related to Subtilisin Carlsberg (CAB56500) from *Bacillus licheniformis*, Subtilisin E from *Bacillus subtilis* (P04189.3), and Subtilisin BPN′ (P00782) from *Bacillus amyloliquefaciens*, which have been widely applied in industry.

In addition, the primary structure of APrBP is similar to those of reported proteases (Supplementary Table 1) (Deng et al., 2010; Joshi and Satyanarayana, 2013; Tsai et al., 1988; van der Laan et al., 1991). Among them, the aliphatic index of APrBP is relatively high, next to the alkaline protease (WP_095239263.1) from *Bacillus clausii*. Generally, a higher aliphatic index indicates a greater abundance of aliphatic amino acids in the enzyme, suggesting that the enzyme has good thermal stability (Bhatt and Singh, 2020).

### 3.2 Structural model of AprBP

The protein sequence of mature AprBP was submitted to the Swiss-Model web server and showed 88.8% identity with the crystal structure of alkaline proteinase, Savinase from *Bacillus lentus* (PDB code:1TK2), which was selected as the template for the 3D model of AprBP.

The AprBP PDB structure was submitted to several servers to validate its quality. The C-alpha atom root-mean-square deviation (RMSD) between the AprBP homology model and the 1TK2 template was 0.071 Å (Supplementary Figure S1). A *Z*-score of −9.99 for the AprBP model, with most amino acid positions being negative as analyzed by the PROSA server, suggested that the model quality is good (Supplementary Figure S2). Based on Ramachandran plot results which determined by PROCHECK program, 92.7% of the amino acids were in the favored regions and 7.3% in the allowed regions (Supplementary Figure S3). The ERRAT quality factor and the Verify-3D scores were evaluated 91.53 and 95.91%, respectively (Supplementary Figure S4). These validation results indicate that the APrBP model exhibited good quality and is highly reliable for further use.

**Figure 2:**
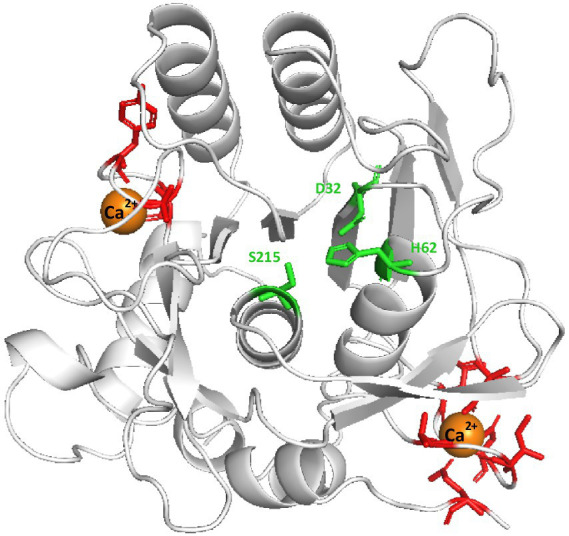
3D structure of mature APrBP protein predicted by Swiss-Model server. The catalytic residues of D32, H62 and S215 are indicated by green sticks. Calcium ion binding sites are depicted as red sticks. The orange balls represent the calcium ions.

APrBP has a compact globular structure primarily composed of nine α-helices and nine β-sheets, with a catalytic site formed by amino acid residues D32, H62, and S215 that are closely positioned to the active site cavity, and two calcium binding sites predicted in this enzyme (Figure 2). The SOPMA tool revealed the second structure consists of 23.79% α-helices, 22.30% β-strands and 53.90% coils, indicating that APrBP is predominantly composed of coils. Coil regions have been reported to improve activity and stability, by enhancing flexibility under saline and alkaline conditions (Zorgani et al., 2014).

### 3.3 Molecular cloning, heterologous expression and purification of APrBP

Based on the genomic sequence of *B. patagoniensis* obtained from NCBI, we designed primers (APrBP *BamHI* F/APrBP *NotI* R) to amplify the gene sequence without the signal peptide by PCR using the *B. patagoniensis* genome as a template by PCR. The amplified fragment (1,134 bp) was then ligated into the pET-28a (+) vector. After verification by sequencing, the recombinant vector pET-28a (+)-APrBP was transferred to *E. coli* BL21 for protein expression. After inducing with 0.1 mM IPTG at 16°C for 24 h, we detected hydrolysis zones through a plate diffusion assay (Supplementary Figure S5). Additionally, an alkaline protease activity of 100.89 U/mg was measured in the intracellular fractions (Table 1), confirming the expression of functional APrBP.

**Table 1:** Purification of alkaline protease APrBP from *E. coli* BL21.

Procedure	Total activity (U)	Total protein (mg)	Specific activity (U/mg)	Purification (fold)	Yield (%)
Crude extract	1255.59	12.44	100.89	1	100
Ni-NTA	572.61	1.19	478.21	4.74	45.61

**Figure 3:**
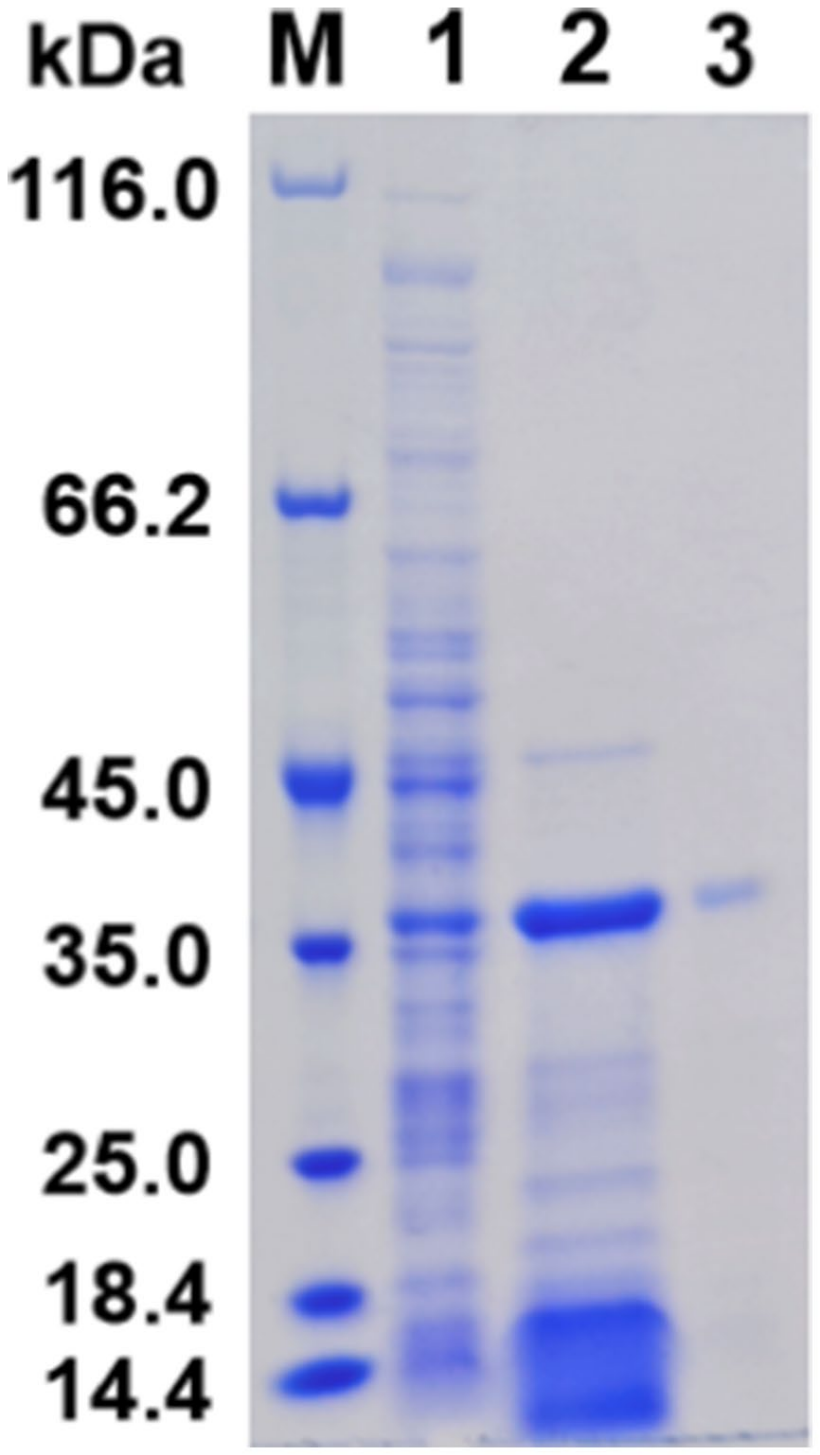
Expression and purification of APrBP. Lane M, protein marker (14.4–116 kDa); lane 1, an uninduced intracellular fraction of *E. coli* cells containing recombinant APrBP; lane 2, an intracellular fraction of *E. coli* cells containing recombinant APrBP induced with 0.1 mM IPTG; and lane 3, the purified APrBP using nickel column affinity chromatography.

SDS-PAGE analysis demonstrated that the induced protein samples exhibited a more intense band at 38 kDa in comparison to the uninduced samples, indicating that this band likely contained the overexpressed APrBP, whose expression was regulated by IPTG induction. Moreover, a significant decrease in other nonspecific bands was observed, suggesting that the overexpressed APrBP might have hydrolyzed certain host proteins (Figure 3).

The harvested cells of *E. coli* (pET-28a (+)-APrBP) transformants were disrupted by sonication in phosphate buffer and purified by nickel column affinity chromatography. The purified enzyme APrBP demonstrated a specific activity of 478.21 U/mg, achieving 4.73-fold purification with a yield of 45.61% compared to the whole crude cell extract (Table 1). A higher yield indicates greater purification efficiency, and the yield of APrBP can be further increased through ammonium sulfate precipitation and heat treatment to fulfill the demands of large-scale production in the future (Jeong et al., 2018; Wen et al., 2022). SDS-PAGE of recombinant APrBP resolved as a single band with a molecular weight of approximately 38 kDa (Figure 3), which corresponded to the expected result. The identification was performed through LC–MS/MS, and after comparing the unique peptide sequences with the database, we found that they were most similar to the sequence WP_078392427.1, which has a similarity of 99% with APrBP. The relevant peptide mass spectra are provided in the Supplementary Figure S6, indicating that APrBP was successfully purified.

### 3.4 Biochemical characterization of APrBP

#### 3.4.1 Effects of pH on activity and stability of APrBP

**Figure 4:**
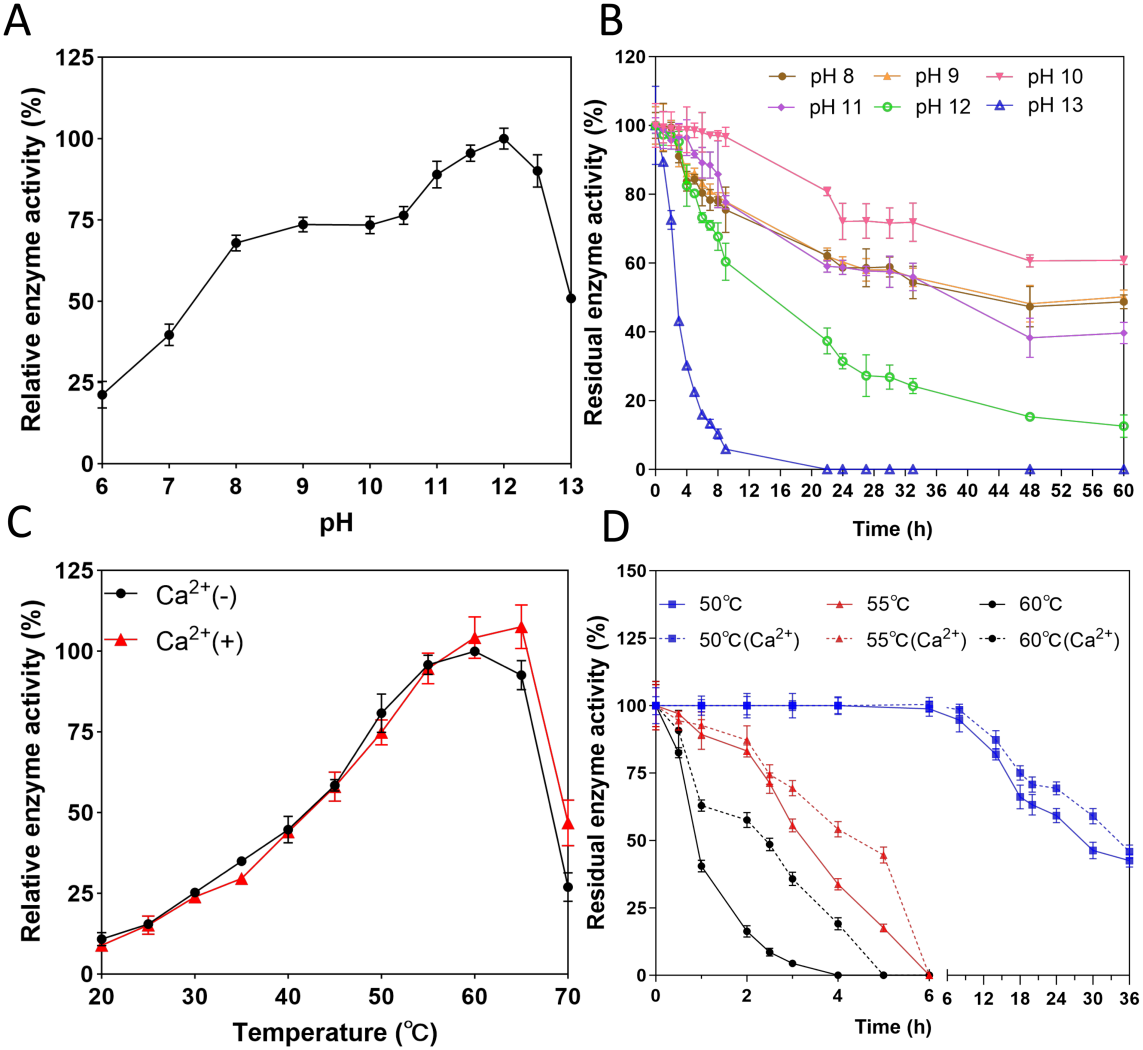
Effect of pH and temperature on APrBP activity and stability. **(A)** Effect of pH on APrBP activity. **(B)** Effect of pH on APrBP stability. **(C)** Effect of temperature on APrBP activity with and without Ca^2+^ addition. **(D)** Effect of Ca^2+^ on APrBP thermostability.

APrBP exhibited enzymatic activity at pH 6–13, with exceeding 50% activity between pH 8 and 13, peaking at pH 12 (Figure 4A). The optimal pH was similar to that of the alkaline protease from *Bacillus gibsonii* (Mahakhan et al., 2023).

As shown in Figure 4B, APrBP was highly stable at pH 8–13. After 60 h of treatment at pH 8, 9, and 10, it retained 48, 50, and 60% of its activity, respectively. The enzyme maintained 55% activity after incubation for 33 h at pH 11. When treated at pH 12 for 9 h, 64% activity was maintained, 43% activity was maintained at pH 13 after treatment for 3 h. The extreme alkalinity tolerance of APrBP surpasses that of most known alkaline proteases, including the reported BcaPRO (Joo et al., 2003) and SAPHM (Hadjidj et al., 2018), as well as the commercial enzymes, Alcalase, Savinase and Subtilisin Novo (BPN′) (Maurer, 2004). This characteristic enables APrBP to function and be stored in highly alkaline environments, highlighting its significant potential for applications in detergents and similar fields.

#### 3.4.2 Effects of temperature on activity and stability of APrBP

The optimum temperature of APrBP was 60°C in the absence of Ca^2+^, and increased to 65°C in the presence of Ca^2+^ while the enzyme activity increased from 27 to 46% at 70°C with Ca^2+^ addition (Figure 4C). Most alkaline proteases have an optimal temperature ranging of 40°C–70°C (Barzkar, 2020). APrBP shares a common optimal temperature of 60°C with the proteases of *B. alkalitelluris* TWI3 (Anandharaj et al., 2016), *B. koreensis* BK-P21A (Anbu, 2013), *Bacillus* sp. SM2014 (Jain et al., 2012) and *Bacillus* sp. B001 (Deng et al., 2010).

APrBP demonstrated significant stability at 50°C, retaining 58% activity after 25 h of heat treatment, and maintaining 59% of its activity for 30 h with the addition of Ca^2+^. However, the residual enzyme activity of APrBP decreased sharply to 40% after incubation at 60°C for 1 h, and significantly increased to 48% after 2.5 h in the presence of Ca^2+^ (Figure 4D). The addition of Ca^2+^improved the optimal temperature and thermal stability of APrBP, due to the presence of two binding sites for Ca^2+^ ions in its structure, a characteristic possessed by most subtilisins (Azrin et al., 2022). Ca^2+^ helps stabilize the enzyme’s conformation by electrostatic interactions (Zhang et al., 2019), which explains why Ca^2+^ was beneficial for improving APrBP activity and stability. APrBP was quite stable at 50°C, making it suitable for long-term fermentation food production and the detergent industry at this temperature.

#### 3.4.3 Effect of mental ions on the activity of APrBP

The effects of metal ions on the APrBP activity were investigated (Table 2). Enzyme activity decreased to 97.1, 79.6, 81.2, and 70.1% after treatment with 10 mM Ba^2+^, Fe^3+^, Fe^2+^, and Co^2+^, respectively. APrBP activity was slightly improved by 10 mM Mg^2+^, Ni^2+^, and K^+^, except for Cu^2+^, which completely inactivated the enzyme at 1 mM. A high concentration of Mn^2+^ (10 mM) significantly promoted the activity of APrBP by 157%, while the addition of Ca^2+^ (10 mM) increased the activity to 113%. Similar promoting effects of Mn^2+^ were reflected on the enzyme activity of the alkaline protease from *Bacillus halotolerans* (Wen et al., 2022). APrBP contains two Ca^2+^ binding sites, and several studies have reported that the addition of Ca^2+^ enhances the activity of alkaline proteases (Li et al., 2023; Raval et al., 2022; Zaman et al., 2023). In contrast, Cu^2+^ has been shown to significantly inhibit the alkaline proteases of certain *Bacillus* species (Kamran et al., 2015).

**Table 2:** Effect of metal irons on the activity of APrBP.

Agents	Relative activity (%)		
	1 mM	5 mM	10 mM
Control	100 ± 3.33	100 ± 2.11	100 ± 1.32
Ca^2+^	106.58 ± 1.86	110.09 ± 4.47	113.60 ± 3.28
Ba^2+^	90.51 ± 4.82	93.85 ± 2.12	97.14 ± 1.22
Mg^2+^	97.77 ± 2.86	102.80 ± 1.81	105.03 ± 2.41
Mn^2+^	97.65 ± 0.80	128.32 ± 4.71	157.81 ± 3.02
Ni^2+^	95.67 ± 3.14	110.37 ± 2.53	108.01 ± 1.82
Fe^3+^	99.22 ± 3.46	88.40 ± 1.92	79.62 ± 3.44
Fe^2+^	67.70 ± 0.98	89.26 ± 2.60	81.29 ± 1.26
Cu^2+^	0 ± 1.41	2.14 ± 4.7	1.60 ± 3.92
Co^2+^	98.28 ± 1.76	92.16 ± 1.98	70.15 ± 4.22
K^+^	90.91 ± 3.34	100.16 ± 3.70	100.97 ± 2.91

#### 3.4.4 Effects of chemical reagents on the activity of APrBP

Stimulatory effects were observed for non-ionic surfactants including Triton X-100, Tween-20 and Tween-80 at all concentration, but anionic surfactant 0.5% SDS exhibited a severe inhibition on enzyme activity which dropped to under 10% (Table 3). Several studies have also revealed the inhibitory effects of SDS on protease activity (Jeong et al., 2018; Li et al., 2022; Wen et al., 2022). Although oxidizing reagents have been found to destabilize many of alkaline proteases (Joo et al., 2003), APrBP retained 75.1% of its activity after incubation with 5% H_2_O_2_. Following pretreatment with reducing agents 5 mM DTT and 5 mM β-mercaptoethanol, APrBP retained 75.5 and 111.3% of its activity, respectively. The alkaline protease from *Bacillus lehensis* also exhibits similar properties (Joshi and Satyanarayana, 2013). These findings indicated that APrBP is resistant to both oxidants and reducing agents.

**Table 3:** Effect of chemical reagents on the activity of APrBP.

Agents	Concentration	Relative activity (%)
Control	—	100 ± 2.95
H_2_O_2_	1%	84.50 ± 5.02
	5%	75.10 ± 5.81
β-Mercaptoethanol	1 mM	117.46 ± 1.7
	5 mM	111.18 ± 5.02
DTT	1 mM	90.25 ± 5.67
	5 mM	75.53 ± 7.35
NaCl	0.5 M	106.57 ± 1.56
	1 M	86.36 ± 3.57
	2.5 M	60.22 ± 2.84
EDTA	1 mM	39.5 ± 2.20
	5 mM	11.41 ± 4.95
PMSF	1 mM	59.32 ± 1.82
	5 mM	8.3 ± 3.31
SDS	0.1%	39.73 ± 2.74
	0.5%	7.53 ± 2.46
TritonX-100	0.5%	128.9 ± 4.80
	1%	125.56 ± 4.59
Tween-20	0.5%	131.17 ± 2.52
	1%	134.81 ± 2.94
Tween-80	0.5%	104.64 ± 5.76
	1%	104.67 ± 3.10

After an hour pre-incubation at 25°C with NaCl (0.5–2.5 M), the APrBP activity increased to 106.57% at 0.5 M, but decreased to 86.36 and 60.22% at 1 M and 2.5 M, respectively. These results are consistent with previous findings that the proteases of haloalkaliphilic *Bacillus* strains were activated at NaCl concentrations ranging from 0.2 to 0.5 M, but were inhibited at higher concentrations (Mokashe et al., 2018).

Additionally, after treatment with 5 mM PMSF and EDTA inhibitors, the enzyme activity decreased to 8.3 and 11.4%, respectively. Inhibition by PMSF indicates that APrBP is a serine protease, as the serine active site is disrupted by PMSF (Wen et al., 2022). EDTA, as a metal chelator, also almost completely inactivated the enzyme, indicating that APrBP activity requires metal ions (Bhatt and Singh, 2020).

#### 3.4.5 Effects of organic solvents on stability of APrBP

Most of the known proteases are unstable in organic solvents, limiting their applications in non-aqueous media (Kikani et al., 2023). The stability of APrBP incubated with diverse organic solvents characterized by log *p*-values between −0.76 and 3.5 was evaluated in this study. Enzyme solutions containing 50% (v/v) organic solvents were shaken at 25°C for 72 h. APrBP exhibited significantly higher stability in various organic solvents, including methanol, acetone, glycerol, DMSO, n-hexane, and ethyl acetate, than the control, with the exception of chloroform (Table 4). The organic solvent stability of APrBP was demonstrated to be superior to that of the commercial enzyme Thermolysin type X, which was significantly inhibited by 50% (v/v) methanol and ethyl acetate (Hadjidj et al., 2018; Omrane Benmrad et al., 2019).

**Table 4:** Effect of organic solvents on APrBP stability.

Organic solvents	Log *p*	Concentration (% v/v)	Residual activity (%)
Control	—	—	100
Methanol	−0.76	50	335.66 ± 2.51
Acetone	−0.24	50	400.63 ± 4.98
Glycerol	−1.76	50	374.52 ± 2.33
DMSO	−1.35	50	245.85 ± 2.89
N-hexane	3.5	50	254.14 ± 2.25
Ethyl acetate	0.68	50	445.85 ± 3.33
Chloroform	1.97	50	5.73 ± 0.31

Organic solvents with log *p* < 4.0 have higher degrees of partitioning into the aqueous layer, substantially disrupting the hydrogen binding and hydrophobic interactions. This disruption reduces the structural flexibility of the enzymes, resulting in significant enzyme inactivation (Jain et al., 2012). Conversely, halophilic bacteria accumulate large amounts of salt within their cells, thereby lowering the water content in the cytoplasm and creating a non-aqueous environment. This adaptation allows for the selection of enzymes that function effectively in such conditions (Kikani et al., 2023; Sarwa et al., 2024; Xiong et al., 2007), potentially explaining the resistance of APrBP to organic solvents. The exceptional stability of APrBP makes it an ideal biocatalyst for peptide synthesis in low-water-activity systems.

#### 3.4.6 Substrate specificity and kinetic study

**Figure 5:**
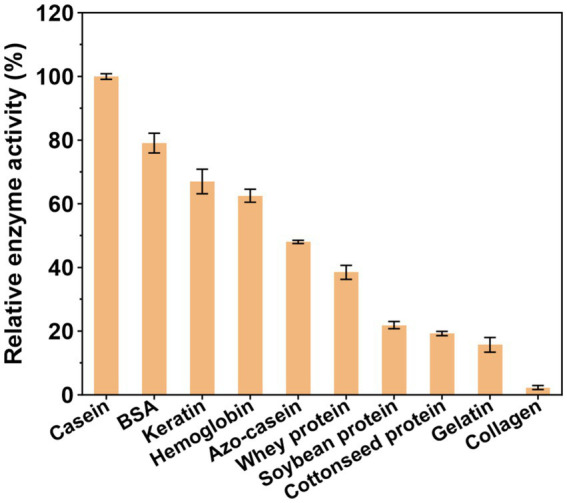
Substrate specificity of APrBP.

APrBP exhibited the highest enzymatic activity towards casein (100%), followed by BSA (79%), keratin (67%), hemoglobin (63%), azo-casein (48%), and whey protein (40%). Contrarily, APrBP demonstrated a weak ability to hydrolyze gelatin, and was almost incapable of hydrolyzing collagen (Figure 5). The substrate specificity resembled that of the salt-stable protease from *Jeotgalicoccus* sp. (casein > BSA > hemoglobin > gelatin) (Mokashe et al., 2015). These results suggested that APrBP has significant potential in the detergent industry and feather degradation, with possible applications in whey protein hydrolysis.

The enzyme kinetics were visualized using a Lineweaver–Burk plot (Supplementary Figure S7). APrBP exhibited classical Michaelis–Menten kinetics when casein was used as the substrate at concentrations of 1–20 mg/mL. The values of *V*_max_, *K*_m_, *K*_cat_, and *K*_cat_/*K*_m_ were 26,553 U/mg, 0.544 mg/mL, 11948.8 s^−1^, 21972.7 s^−1^ (mg mL^−1^)^−1^, respectively. Additionally, the *K*_cat_*/K*_m_ of the alkaline protease BaApr1 from *Bacillus altitudinis* W3 was 15926.07 s^−1^ (mg mL^−1^)^−1^ towards casein (Yang et al., 2020). The higher of *K*_cat_*/K*_m_ of APrBP indicates that it possesses high catalytic efficiency and affinity towards casein, thereby supporting its industrial applications.

#### 3.4.7 Substrate docking simulation

**Figure 6:**
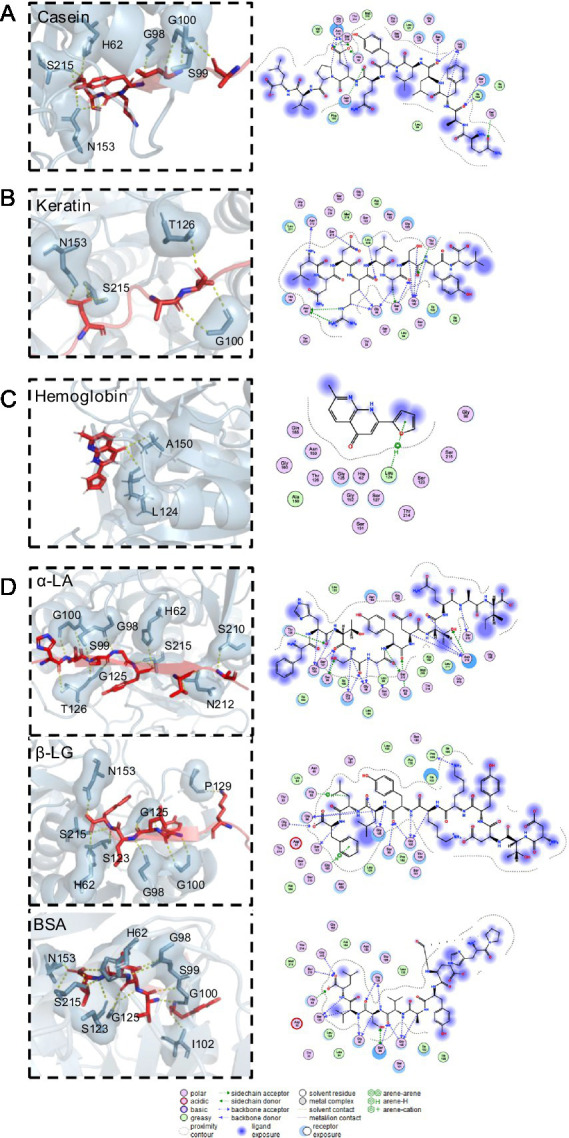
Molecular docking results for **(A)** APrBP-casein. **(B)** APrBP-keratin **(C)** APrBP-hemoglobin and **(D)** APrBP-α-LA, APrBP-β-LG, and APrBP-BSA. The left side shows the 3D structure of the peptide, with the red stick structure representing the substrate peptide. The yellow dashed lines indicate hydrogen bonds, highlighting the interactions between molecules. The blue stick structure represents APrBP. Key compounds are represented by sticks, while other proteins are depicted as cartoons. The right side presents the 2D structure of APrBP.

Molecular docking was used to elucidate the binding patterns of the small molecular substrates and APrBP, aiming to predict their conformations and binding affinities. The docking results of small molecules, including casein, keratin, hemoglobin and three whey proteins, namely α-LA, β-LG and BSA, with the binding sites of alkaline protease are depicted in the Figure 6. Their binding affinities to APrBP were −9.4, −5.0, −5.6, −5.7, −9.1, and −8.1 kcal/mol, respectively. The binding energy reflects the strength of the interaction between the enzyme and the substrate. A lower binding energy indicates a more stable structure formed by the enzyme-substrate complex (Mohammadzadeh-Asl et al., 2020).

A thorough examination revealed that casein was positioned within the active pocket (Figure 4A), engaging with active-site residues (H62, N153, and S215) and additional amino acid residues (G98, G100, I102, and T214) through hydrogen bonds. The 2D diagram of the protein-peptide complex revealed that 11 active pocket amino acid residues (L94, S99, S101, S123, L124, G125, T126, L133, A150, G152, and P162) were interacting with the casein peptide substrate through hydrophobic interactions, van der Waals interactions and hydrogen bonding.

The keratin peptide interacted with active center of the protease, forming hydrogen bonds with the active-site residue S215 and active pocket amino acid residues (N60, G100, I102, G125, and T214) (Figure 6B). Hemoglobin could be fully embedded into active pocket of the protease and tightly bound to the protease’s active site, L124, through hydrogen bonds (Figure 6C). It has been reported that non-covalent hydrogen bonds can enhance the binding of enzymes to substrates, and low-barrier hydrogen bonds are crucial for improving the catalytic efficiency of serine proteases (Yan et al., 2025).

Moreover, α-LA, β-LG, and BSA formed hydrogen bonds with the APrBP catalytic sites (H62 and S215) and other active pocket residues (G98, S99, G100, and S123). In addition to hydrogen bonding and hydrophobic effects with these peptides, H62 formed salt bridges with aspartic acid in α-LA, phenylalanine in β-LG, and leucine in BSA, further improving the stability of the peptide-protease binding (Figure 6D).

Casein, keratin, hemoglobin and whey proteins formed stable interactions with the protease APrBP, positioning themselves in the active pocket of APrBP during binding and displaying favorable binding poses. Consequently, the protease can efficiently hydrolyze these substrates, laying the foundation for their application in detergents, whey protein hydrolysis, and feather degradation.

### 3.5 Applications of the recombinant APrBP

#### 3.5.1 Stability of APrBP and the washing performance in detergents

Proteases and other enzymes (e.g., lipases and amylases) have become essential components in detergents. Besides enzymes, detergents also contain 0.004% ionic surfactants, 0.03% non-ionic surfactants, 0.02% bleaching and oxidizing agents, 0.4–0.8% sodium chloride, and various other chemical additives, including disodium cocoamphodiacetate and cyclodextrin (Akram et al., 2020; Blyth et al., 2013; Mokashe et al., 2017, 2018). It is crucial to ensure that proteases remain stable in the presence of these components to effectively perform their cleaning function effectively.

**Figure 7:**
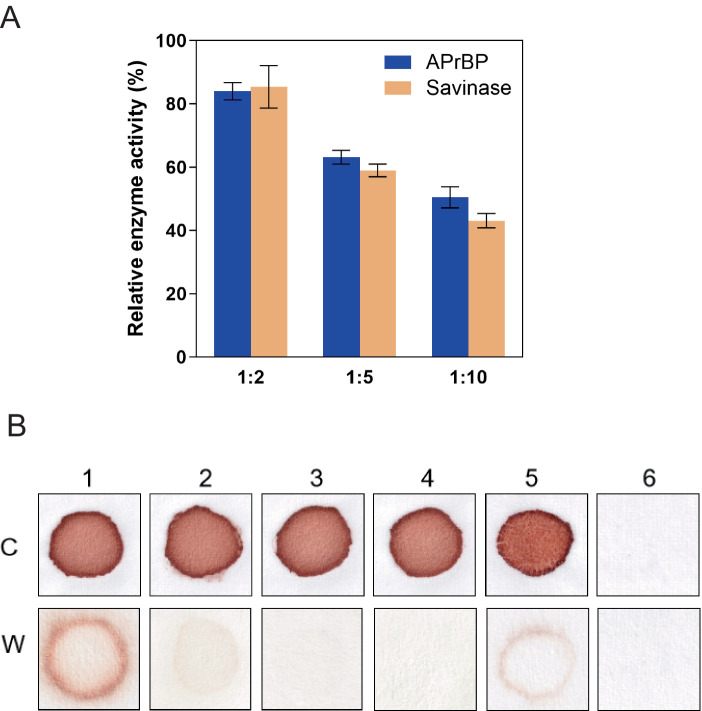
Stability of APrBP in laundry detergent solution and washing performance of APrBP. **(A)** Stability of APrBP and Savinase^™^ in the presence of heat-treated detergent. The enzymes were diluted with detergent solution three-fold (1:2), six-fold (1:5) and eleven-fold (1:10). The enzyme activity with no addition of laundry detergent was taken as 100%. **(B)** Cloth stained with blood washed with 1, tap water; 2, heat-treated detergent; 3, non-heat treated detergent; 4, heat-treated detergent and APrBP; 5, APrBP; 6, unstained cloth washed with tap water, C, Cloth before washing. W, washed clothes.

To evaluate the stability of APrBP in the laundry solution, the enzyme was incubated with heat-treated laundry solutions at various dilution ratios at 40°C for 30 min, with Savinase^™^ as a reference. The results indicated that a higher dilution factor corresponded to a greater loss of enzymatic activity (Figure 7A). Specifically, at a dilution ratio of 1:5, APrBP retained 63.1% of its activity, while Savinase^™^ maintained 58.9%. At a dilution of 1:10, APrBP’s activity decreased to 50.4%, compared to 43.1% for Savinase^™^. The stability of APrBP in laundry solutions surpasses that of Savinase^™^, a commercial protease applied in the detergent industry (Sarwa et al., 2024).

Due to its high activity and stability of APrBP at pH 8–13, as well as its strong substrate specificity for hemoglobin, the application of APrBP as a detergent additive for blood stains was evaluated. When crude enzyme, APrBP (50 U/mL) was added to heat-treated Tide solution (7 mg/mL), its washing efficacy was identical to that of the non-heat treated Tide solution, both achieving complete removal of blood stains. However, washing with only tap water, heat-treated Tide solution or the enzyme APrBP failed to remove the stains completely (Figure 7B). Proteases derived from various microorganisms are increasingly being used for blood stain (Akram et al., 2020; David et al., 2018; Hemsinli and Gurkok, 2024; Rekik et al., 2019). Compared to alkaline proteases at 50–90 U/mL (Hammami et al., 2017; Paul et al., 2014; Ramakrishna Reddy et al., 2017), APrBP at 50 U/mL completely removed blood stains within 10 min, showing its high cleaning efficiency. These results demonstrated that APrBP could serve as a useful bio-additive in laundry detergents.

#### 3.5.2 Feather degradation

Feathers contain 80–90% crude protein, mainly keratin, making them a valuable protein resource (Huang et al., 2019). Currently, researchers have shown great interest in using microbial enzymes to degrade feathers (Rahimnahal et al., 2023). Herein, the potential application of APrBP in feather waste recycling was evaluated by measuring the feather degradation rate and components of the feather hydrolysate.

**Figure 8:**
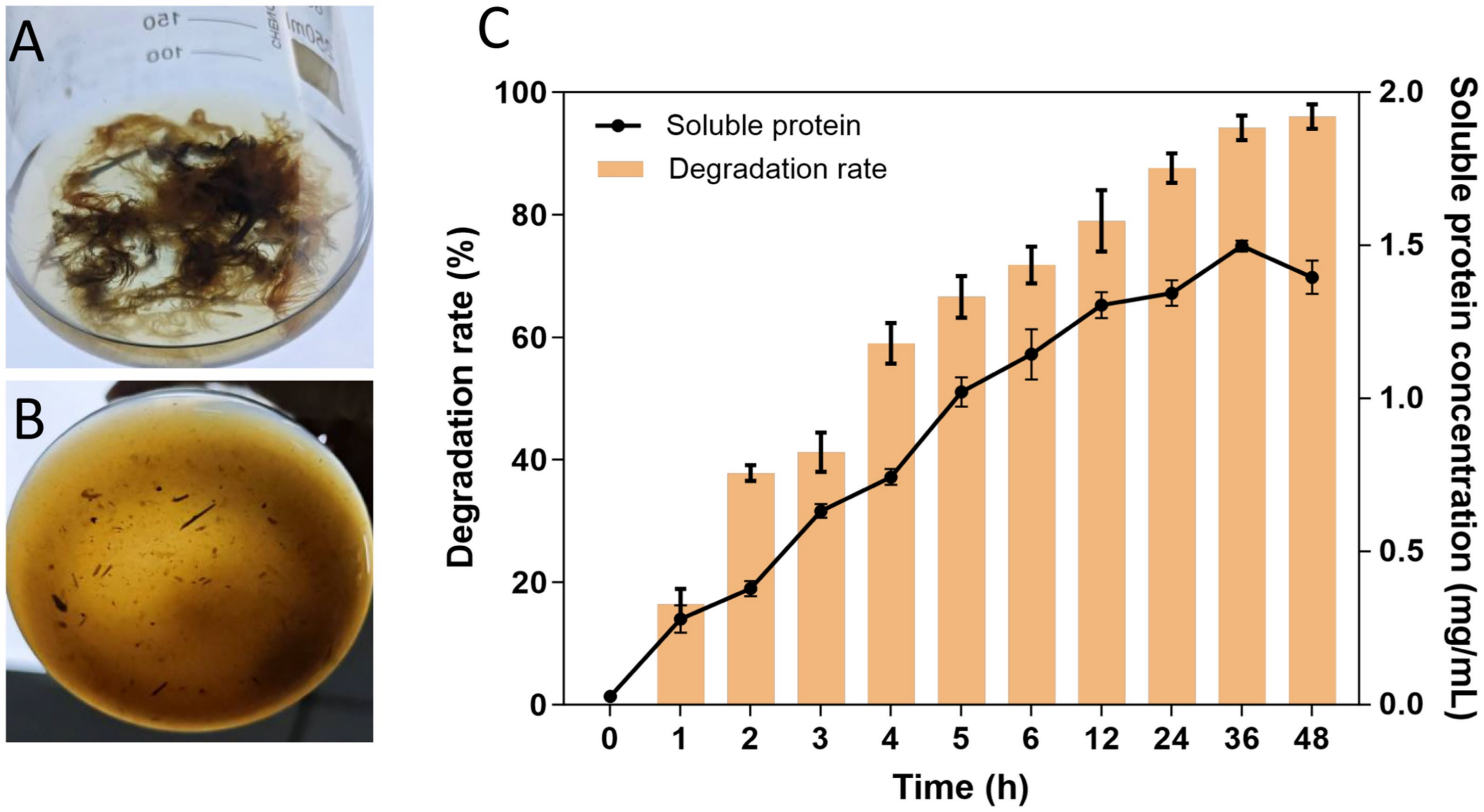
Efficiency of feather degradation. **(A,B)** 1% feather culture medium containing recombinant APrBP (1,000 U) at 0 and 48 h, respectively. **(C)** Feather degradation rate of 1% feather culture medium (bar graph) and soluble protein concentration in the feather culture medium (line graph).

The APrBP crude enzyme (20 U/mL) was added to 1% (w/v) FB medium and incubated at 50°C. APrBP could completely degrade feathers within 48 h (Figures 8A,B). The degradation rate reached 88% after 24 h, with nearly complete hydrolysis (96%) after 48 h (Figure 8C). The soluble protein content peaked at 1.49 mg/mL after 36 h, and then decreased (Figure 8C), possibly due to the decomposition of soluble proteins into small amino acids. After 48 h of fermentation, the feather hydrolysate contained 681.8 mg/L of total free amino acids, primarily phenylalanine, serine, valine, alanine, glycine, and tyrosine (Table 5). Phenylalanine and valine are essential amino acids, while tyrosine is non-essential (Peng et al., 2019). These results demonstrated that the feather hydrolysate contains large quantities of soluble proteins and various free amino acids, indicating its applications in the feed, fertilizer and biomedical industries (El Salamony et al., 2024; Liang et al., 2021). This study preliminarily demonstrated that APrBP was effective in hydrolyzing feathers. However, there is an urgent need in the industry for biocatalysts that can significantly degrade high concentrations of feathers. It has been reported that whole-cell catalysis is more effective in hydrolyzing feathers compared to the addition of enzyme solution alone (Liang et al., 2021). Therefore, overexpressing APrBP in *Bacillus subtilis* and using this host for whole-cell catalysis in feather hydrolysis may be a viable approach.

**Table 5:** Analysis of free amino acids in feather hydrolysate.

Amino acid	Concentration (mg/L)	
	Inactive APrBP (48 h)	APrBP (48 h)
Aspartic acid	1.088 ± 0.11	30.766 ± 1.05
Glutamate	1.044 ± 0.15	14.968 ± 1.32
Serine	ND	101.62 ± 4.89
Glycine	4.098 ± 0.28	55.643 ± 5.03
Threonine	1.153 ± 0.21	33.34 ± 3.54
Arginine	ND	46.055 ± 2.55
Alanine	ND	68.66 ± 4.21
Tyrosine	5.087 ± 0.35	52.15 ± 2.89
Valine	ND	84.23 ± 3.79
Methionine	ND	0.964 ± 0.11
Phenylalanine	5.104 ± 0.57	123.53 ± 1.59
Isoleucine	ND	19.22 ± 1.35
Leucine	ND	34.11 ± 2.67
Lysine	ND	4.831 ± 0.68
Cystine	ND	10.80 ± 1.21
Histidine	ND	0.94 ± 0.09

#### 3.5.3 Whey protein hydrolysis

Although whey protein is a safe and high-quality protein source in itself, it is not suitable for individuals with milk protein intolerance or poor digestive function. Moreover, the beneficial properties of peptides are lost during gastrointestinal digestion. For instance, long-chain peptides may be degraded by brush border enzymes or cell peptidases, leading to a loss of activity, while low molecular weight peptides can be preserved and continue to their function in the tissue (Guo et al., 2019). Consequently, whey protein hydrolysates (WPH) better retain low-molecular-weight bioactive peptides, improving their bioavailability and making them appropriate for a larger population. Bio-active peptides from WPH have been demonstrated some physiological functions, including antibacterial, antihypertensive, and antioxidant properties (Irazoqui et al., 2024). For example, due to the high content of sulfur-containing amino acids in whey protein, WPH contains a higher concentration of antioxidant peptides (Zhao and Ashaolu, 2020). These antioxidant peptides can inactivate reactive oxygen species and scavenge free radicals, thereby delaying the progression of cardiovascular diseases (Lermen et al., 2023).

**Figure 9:**
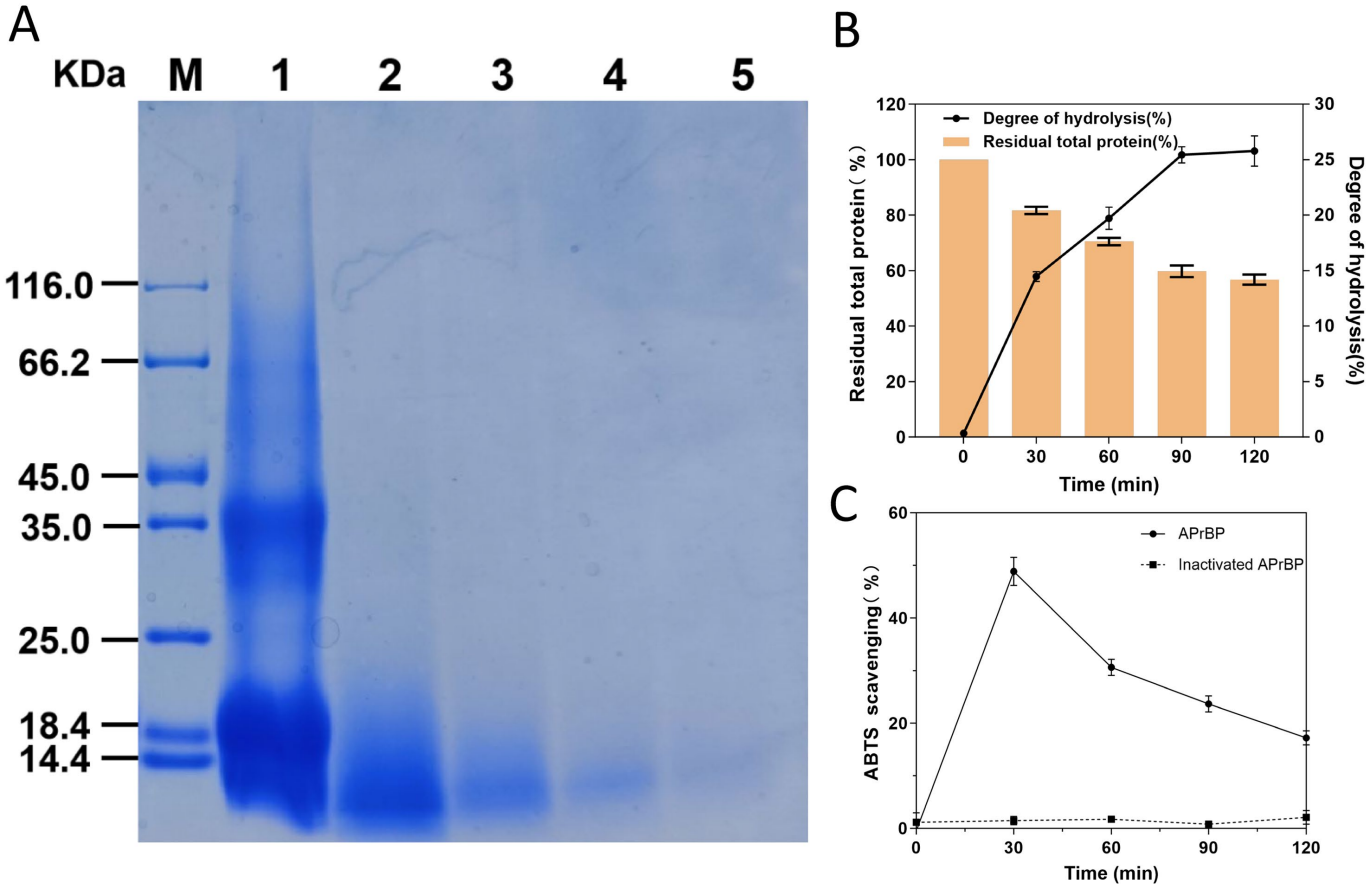
Hydrolysis of whey protein by APrBP protease. **(A)** 15% SDS-PAGE of whey proteins hydrolyzed with APrBP. **(B)** Effects of APrBP on the total protein and degree of hydrolysis of whey protein. The bar graph represents the percentage of total residual protein and the line graph corresponds to the degree of hydrolysis. **(C)** The antioxidant ability of WPH measured using ABTS radical scavenging assay.

The hydrolysis of whey protein using APrBP is depicted in Figure 9. APrBP gradually hydrolyzed large molecular weight proteins into smaller peptides in a time-dependent manner over the course of 120 min (Figure 9A). The total protein content decreased to 59%, and the degree of hydrolysis reached 25.4% in 90 min, after which it began to stabilize (Figure 9B). The degree of hydrolysis (DH) reflects the maximum extent of the enzymatic hydrolysis, and it ranges from 5 to 23% within 6–8 h in most case (Lermen et al., 2023; Sinha and Khare, 2015). A 29.33% hydrolysis of whey protein was achieved using pepsin, while ultrasound and ohmic heating pretreatment and pepsin resulted in 37.74% hydrolysis (Alizadeh and Aliakbarlu, 2020). These results indicated that APrBP can effectively hydrolyze whey protein in a shorter time.

Furthermore, the antioxidant ability of WPH was measured using the ABTS decolorization assay. The results demonstrated that the hydrolysate obtained after 30 min of hydrolysis achieved the maximum ability to scavenge ABTS free radicals at 48.5%. Conversely, the whey protein added with inactivated APrBP demonstrated no free radical scavenging activity indicating that the peptides released from whey protein hydrolysis exhibited antioxidant capacity. The ability to scavenge ABTS free radicals began to decline after 30 min, which may be due to the hydrolysis of some antioxidant peptides (Figure 9C).

Our study demonstrated that the hydrolysis of whey protein with APrBP is a promising strategy for preparing WPH with high antioxidant activity. These results indicate that adding APrBP to whey protein is advantageous, as it increases the protein’s value and enhances its absorption.

## 4 Conclusion

We heterologously expressed a novel alkaline protease, APrBP from *Bacillus patagoniensis*, which showcases attractive properties. The purified recombinant APrBP exhibited maximum activity at pH 12.0 and 60°C. The APrBP protease not only demonstrated extreme alkalinity tolerance but also maintained good activities in the presence of both oxidizing and reducing agents, making it suitable for detergent conditions. APrBP exhibited a strong resistance to high concentration (50%, v/v) of organic solvents, highlighting its potential for non-aqueous biocatalysis. The enzyme has strong substrate specificity towards various natural proteins, including casein, BSA, hemoglobin, keratin, and whey proteins. Molecular docking was employed to understand the interaction mechanisms between APrBP protease and its substrates. The application of APrBP as a detergent formation revealed that APrBP was more stable in detergent solutions than Savinase^™^, and could wash the blood stains with high efficiency. Moreover, APrBP was used to degrade poultry feathers, achieving an 96% degradation rate for 1% of chicken feathers. The hydrolysates contained abundant soluble proteins and free amino acids which are beneficial for animal feed, cosmetics, and pharmaceuticals. Additionally, APrBP demonstrated an advantage in the rapid hydrolysis of whey proteins. To achieve the industrial application of APrBP, further research should address two key issues. First, it is necessary to enhance the stability of APrBP at high temperatures through directed evolution and rational design. Second, the focus should shift from flask fermentation to fermenter fermentation to explore the optimal conditions for the large-scale production of APrBP.

## Data Availability

The alkaline protease gene (aprBP) sequence of Bacillus patagoniensis DB-5 has been submitted to GenBank (Accession No. PV031685).

## Supplementary material

Supplementary FIGURE S1 Root-mean-square deviation (RMSD) diagram for C-alpha atom between the APrBP model (blue) and the template PDB ID: 1TK2 (Magenta).

Supplementary FIGURE S2 Overall APrBP homology model quality validated by the PROSA server.

Supplementary FIGURE S3 Ramachandran graph of AprBP homology model analyzed by the PROCHECK server.

Supplementary FIGURE S4 ERRAT **(A)** and VERIFY3D **(B)** analysis of AprBP homology model.

Supplementary FIGURE S5 Plate diffusion assay using intracellular fraction showing the production of APrBP in *E. coli* BL21.

Supplementary FIGURE S6 LC-MS/MS identification spectrum of the purified APrBP. **(A)** Unique peptide 1, **(B)** Unique peptide 2.

Supplementary FIGURE S7 Kinetic analysis of APrBP visualized by Lineweaver-Burk plot.
