# Supplementary material for: Redox and solvent-stable alkaline serine protease from Bacillus patagoniensis DB-5: heterologous expression, properties, and biotechnological applications
Source: Front Microbiol. 2025 Mar 19;16:1558419. doi: 10.3389/fmicb.2025.1558419 (PMC11970705; doi:10.3389/fmicb.2025.1558419)

**Supplement Table 1**

**Supplement Table 1 Primary structure of APrBP and other reported proteases**

| Enzyme | Asp + Glu/Arg + Lys | Theoretical pI | Aliphatic index | Instability index | GRAVY index |
| --- | --- | --- | --- | --- | --- |
| APrBP | 2.4 | 4.53 | 87.04 | 30.96 | -0.077 |
| AFK08970.1 | 2.19 | 4.54 | 85.64 | 25.59 | -0.037 |
| P20724.1 | 2.19 | 4.49 | 85.64 | 25.51 | -0.049 |
| ADK62564.1 | 4.58 | 3.90 | 80.72 | 36.32 | -0.211 |
| WP_095239263.1 | 2.53 | 4.53 | 87.93 | 35.60 | -0.083 |

**Figure S1**


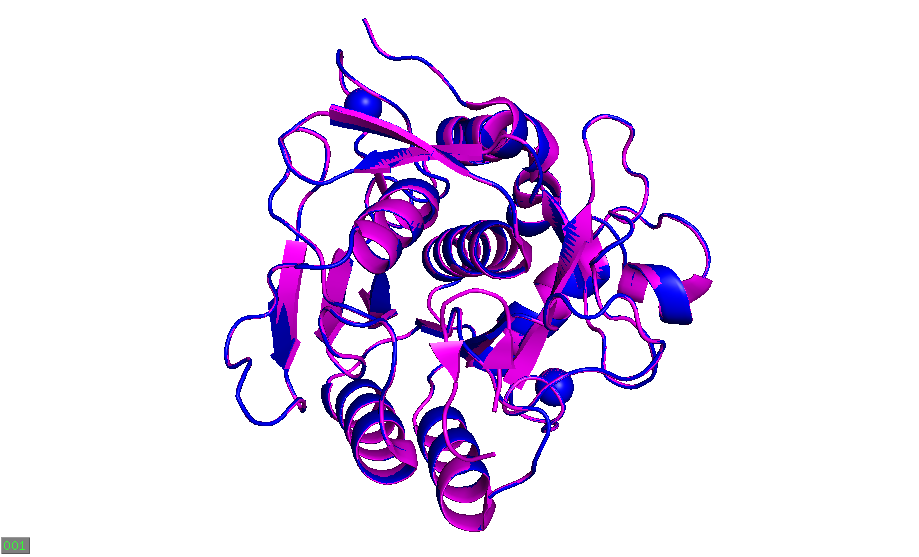


**Figure S2**


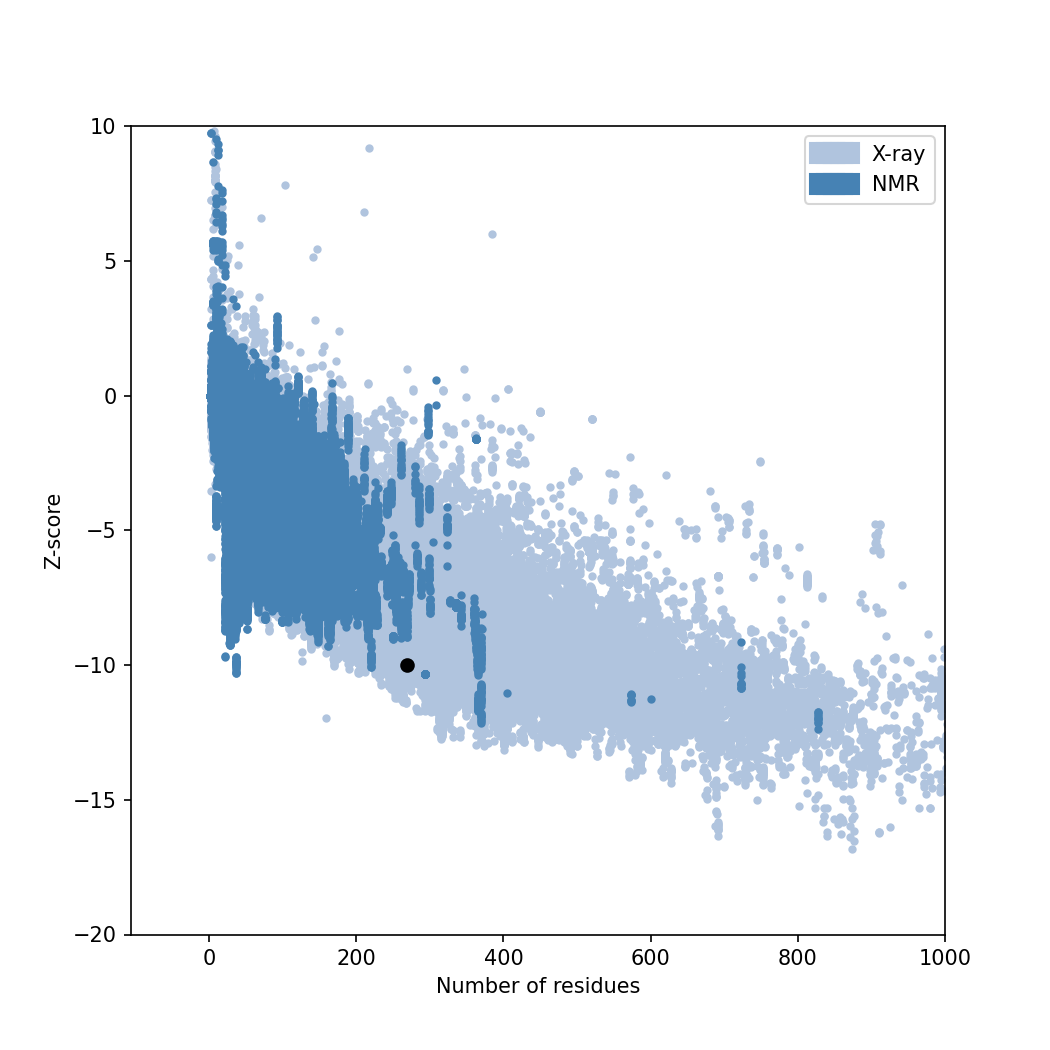


**Figure S3**


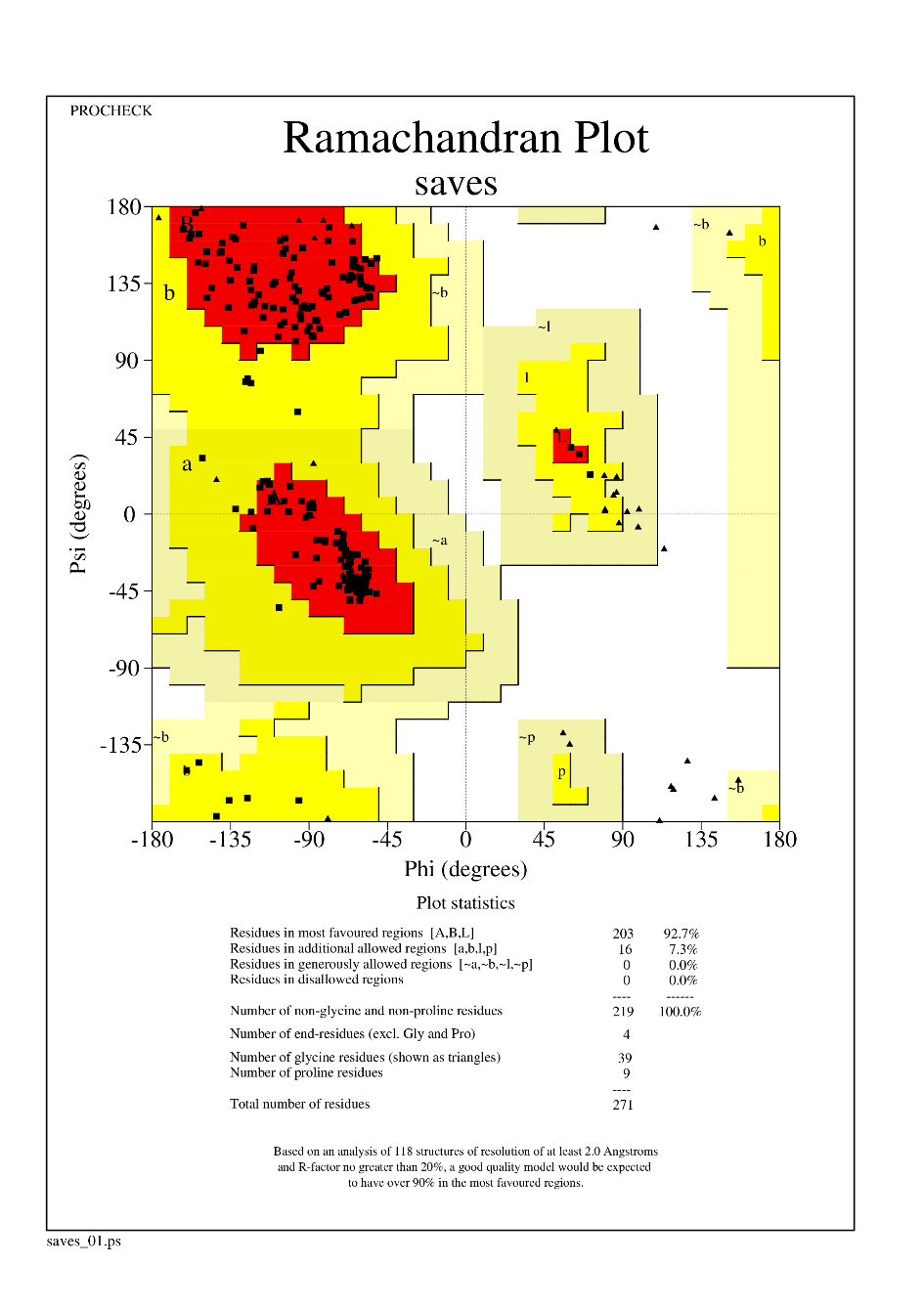


**Figure S4**

A


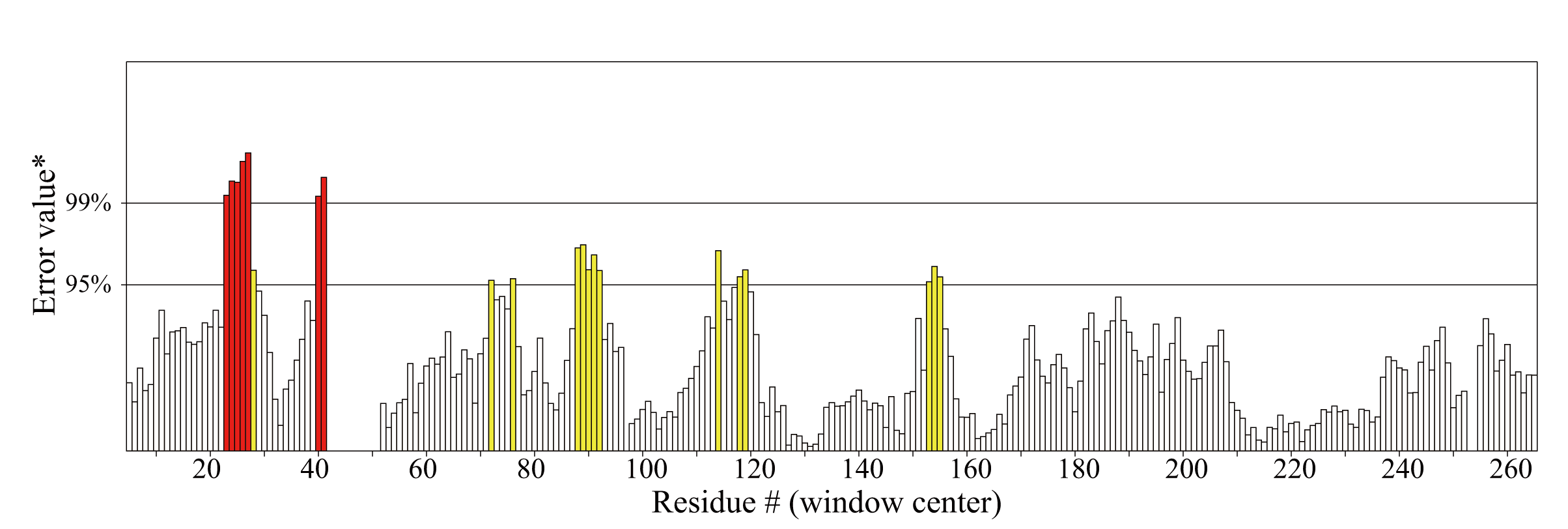


B


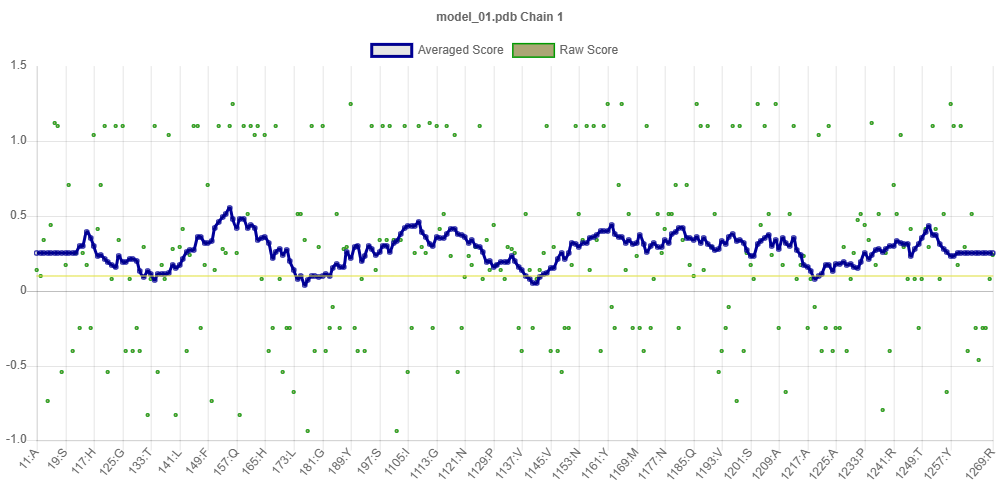


Amino acid sequence of the purified recombinant APrBP

AEEAKEKYLIGFTEQEAVSTFVEQIEEEEVSISEVDDVEIDLLYEFETIPVLSVELNPEDVASLESDPAISYIEEDAEVTTMAQSVPWGISRVQAQSAHNRGITGSGVKVAVLDTGISTHEDLNVRGGASFVAGEPGYQDGNGHGTHVAGTIAALNNSIGVLGVAPNAELYAVKVLGASGSGSISGIAQGLQWAGNNGMHIANMSLGTSAPSATLEQAVNAATAQGVLVIAASGNSGAGSVGYPARYANAMAVGATDQNNNRASFSQYGAGLDIVAPGVGVQSTYPGNRYASLNGTSMATPHVAGVAALVKQKNPSWSNVQVRNHLKNTATNLGNTNLYGSGLVNAEAATREFKLVDLQSRHHHHHH

**Figure S5**


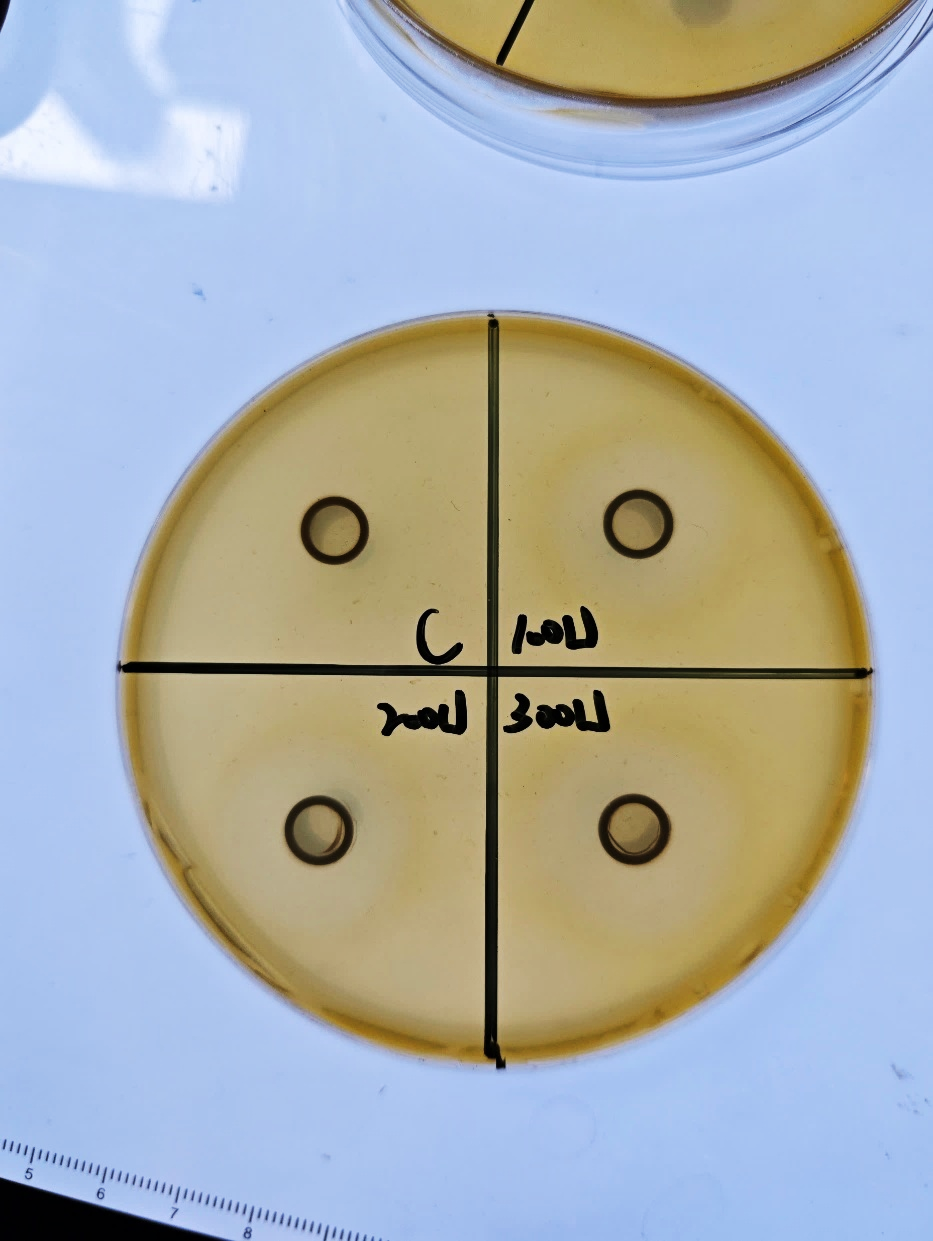


**Figure S6**


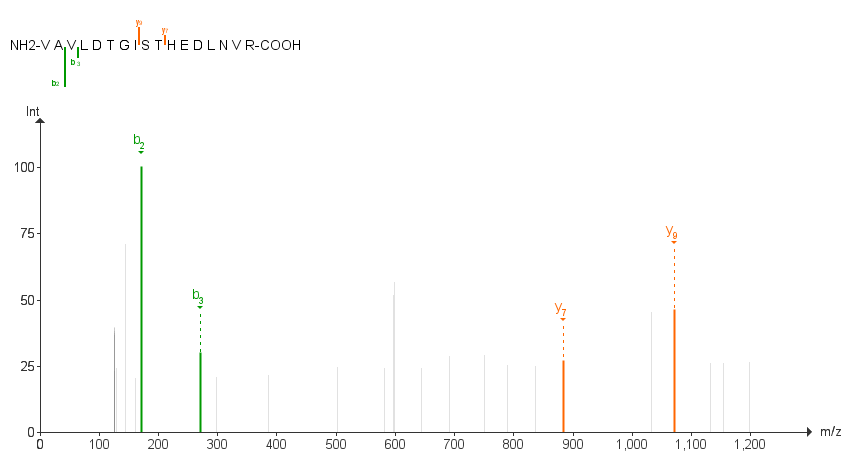
A


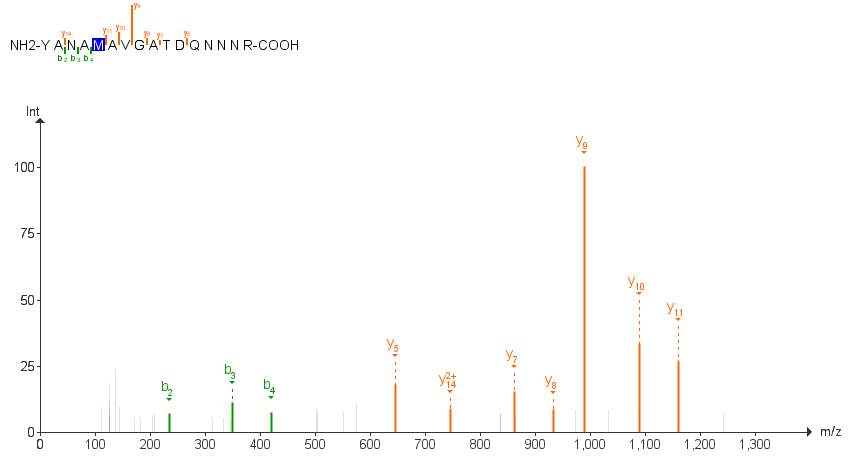
B

**Figure S7**


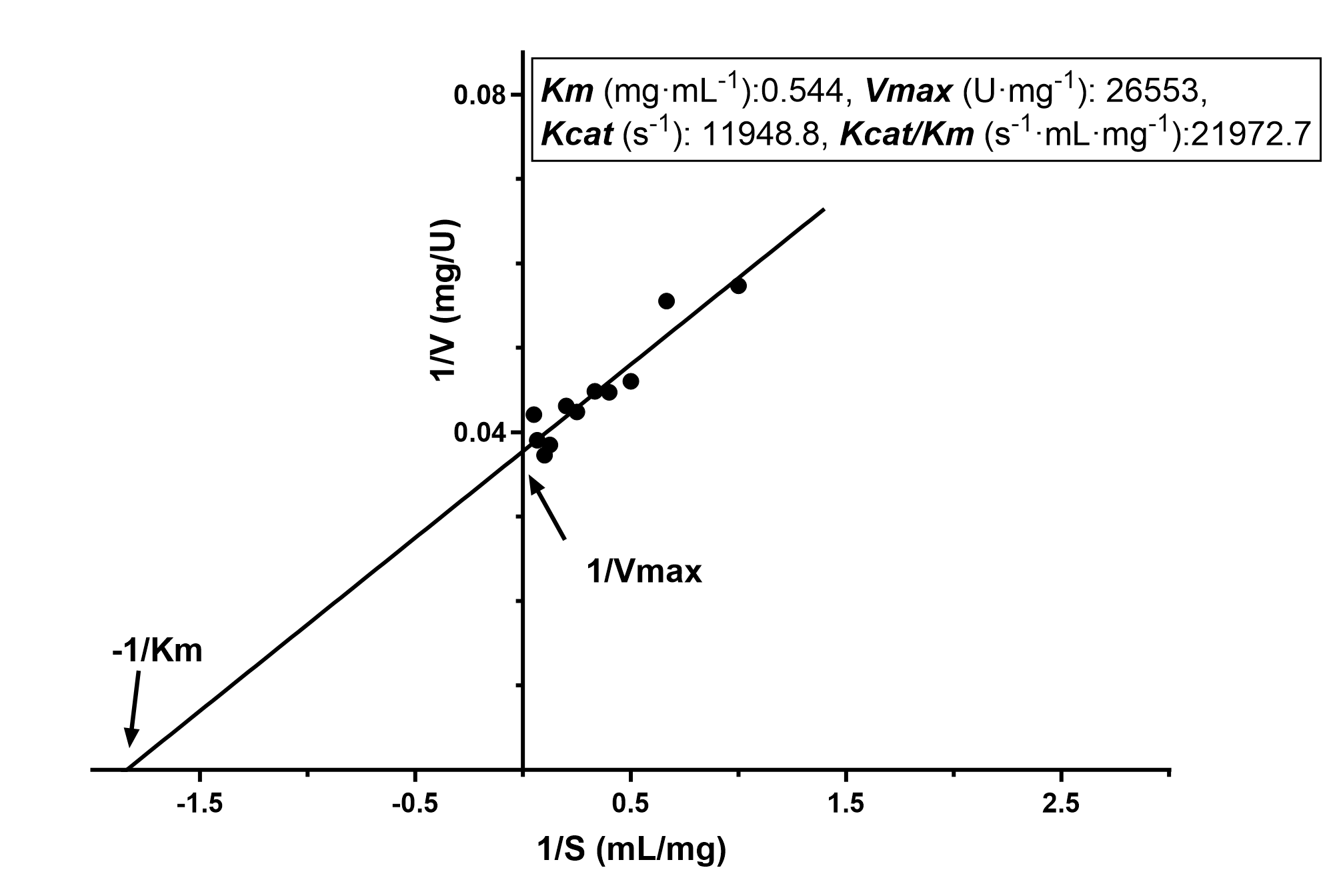

Supplement: Supplementary FIGURE S1 — Root-mean-square deviation (RMSD) diagram for C-alpha atom between the APrBP model (blue) and the template PDB ID: 1TK2 (Magenta). [file Data_Sheet_1.docx]
